# Prevalence of non-communicable diseases and associated medication use among Syrian refugees in Lebanon: an analysis of country-wide data from the Sijilli electronic health records database

**DOI:** 10.1186/s13031-021-00411-3

**Published:** 2021-10-18

**Authors:** Shadi Saleh, Lina Abdouni, Hani Dimassi, Dana Nabulsi, Ranime Harb, Zeinab Jammoul, Noha Hachach, Nour El Arnaout

**Affiliations:** 1 Global Health Institute, American University of Beirut, Beirut, Lebanon; 2 Department of Health Management and Policy, Faculty of Health Sciences, American University of Beirut, Beirut, Lebanon; 3 School of Pharmacy, Lebanese American University, Beirut, Lebanon; 4 American University of Beirut Medical Center, Beirut, Lebanon

**Keywords:** Non-communicable diseases, Chronic diseases, Syrian refugees, Displaced populations, Lebanon, Migration, Hypertension, Diabetes, Cardiovascular diseases, Cancer

## Abstract

**Background:**

Globally, the number of forcibly displaced individuals has reached 70.8 million. Lebanon, a middle income country, hosts the highest number of refugees per capita worldwide. The majority of refugees are Syrians who have fled the Syrian war that started in 2011. The migration journey exposes refugees to increased susceptibility to a wide range of medical issues including non-communicable diseases (NCDs). This study aims to determine the prevalence of NCDs among adult Syrian refugees in Lebanon, with a focus on hypertension, diabetes, cardiovascular diseases (CVD) and cancer. The study also aims to explore factors potentially related to the prevalence figures and understand the medication use associated with these morbidities.

**Methods:**

This study is a secondary analysis of de-identified data from the “Sijilli Electronic Health Records for Refugees” Database comprising data on 10,082 Syrian refugees from across informal tented settlements located all over Lebanon. A total of 3255 records of Syrian refugees aged above 18 years old and reporting having at least one condition of the following were included in the analysis: hypertension, diabetes, cardiovascular diseases or cancer. Pearson’s Chi-square, independent t-test, and multivariate logistic regressions were used for data analysis.

**Results:**

Hypertension was the most prevalent (10.0%) NCD among refugees, and a higher age was associated with higher NCDs prevalence. A strong linkage has been reported between smoking status and alcohol intake, and increased risk for NCDs. Study findings also revealed that the hypertension, diabetes and CVDs were mainly observed among refugees originating from Idlib, Aleppo and Homs. An association between medication use and location of diagnosis was noted, with females who were diagnosed before moving to Lebanon being more likely to take corresponding medications compared to those diagnosed in Lebanon, with no difference reported among males.

**Conclusions:**

Our findings suggest that efforts should be directed towards the employment of innovative low-cost approaches for NCD detection and control among refugees, with a focus on the importance of use of adequate medication. Such efforts remain imperative to control the increasing burden of NCDs amongst refugee populations and improve equitable access to NCD services.

## Background

### The global refugee status

Globally, the number of forcibly displaced individuals including refugees has reached the highest levels on record [1]. In 2019, the United Nations High Commissioner for Refugees (UNHCR) indicated that 70.8 million individuals have been forcibly displaced, among these are nearly 26 million refugees [1]. Although inconsistent [2], the highest reported number of refugees has been hosted by low- and middle-income countries (LMICs). The majority of refugees are of Syrian nationality [3] who have fled since the eruption of the Syrian conflict in 2011 to Syria’s neighboring countries-namely Lebanon, Jordan, Egypt, Turkey and Iraq which are currently heavily burdened, hosting nearly 95% of the total number of registered Syrian refugees worldwide [4]. With such unprecedented figures, the Syrian crisis has been widely described as ‘one of the biggest and worst humanitarian crises of our time' [5].

### The context of Lebanon

Lebanon, an LMIC with a population of over 4 million [6], has become the country with the highest concentration of refugees per capita worldwide with around 1.5 million Syrian refugees [7]. More than half of them are women and children who are in urgent need for healthcare services [8]. Many Syrian refugees have been settled in the poorest and most underserved areas in Lebanon creating social tensions and competition for access to basic healthcare services [9, 10]. This, in turn, overburdened the Lebanese healthcare system which already suffers from limited capacities characterized by insufficient staff, medications and equipment [6]. An estimated 50% increase in healthcare services utilization was reported as a result of the influx of Syrian refugees to Lebanon [11]. Of specific note, a two-fold increase in services occurred at the primary healthcare level related to vaccination, management of communicable diseases, and non-communicable diseases (NCDs), including hypertension, cardiovascular diseases (CVDs), diabetes, chronic respiratory diseases and arthritis [11].

Joint efforts have been noted among different stakeholders (e.g. Lebanese Ministry of Public Health (MoPH), UNHCR, non-governmental organizations (NGOs), etc.) to respond to the health needs of refugee populations and to ensure the widest possible coverage of services [12]. Yet, these efforts were suboptimal [13] due to several impediments [14], including inability to pay out-of-pocket costs, and lack of knowledge about available healthcare services being reported as the major barriers to access among refugees [15]. Nevertheless, refugees who access health services often resort to primary healthcare centers operated by NGOs, or governed by the MoPH to receive services at a discounted cost or free of charge.

### Burdens and aspects of non-communicable diseases (NCDs) among refugees in Lebanon

Non-communicable Diseases (NCDs) are the leading causes of morbidity and mortality worldwide [16, 17]. Globally, over 70% of annual deaths are attributable to NCDs, mainly CVDs, diabetes, cancers, chronic respiratory diseases and cerebrovascular diseases [18, 19]. The burden of NCDs is unevenly distributed between high-income countries (HICs) and LMICs, with around 85% of premature deaths occurring in LMICs [19]. Evidence suggests that NCDs emerge among the top prevalent health conditions in refugees [20, 21]. Moreover, high rates of NCDs have been reported among conflict affected countries receiving large number of refugees, with percentages of concomitant deaths reaching 89% in Lebanon as of before 2018 [19]. Studies found a high prevalence of reported hypertension, diabetes, CVDs and other NCDs amongst Syrian refugees in Lebanon [22, 23], being most pronounced among adults above 40 [24]. One of the most frequently cited nationwide study of NCDs among Syrian refugees in Lebanon in 2016 [25] indicated that the prevalence among refugees reached 7.4% for hypertension, 3.8% for chronic lung diseases, for 3.3% diabetes and 7.9% for arthritis. Moreover, around 77% of total deaths in Syria in 2010 were attributable to NCDs, with CVDs alone being responsible for 44% of total deaths [26].

It is hypothesized that stress, acquired as a result of displacement [27, 28], shortages in medication supplies [29], poor chronic diseases management, and improper lifestyle habits could be recognized as potential contributing factors to the occurrence and worsening of NCDs in refugees [30, 31]. Unfortunately, very little is being done to control and manage these costly diseases among the Syrian refugee population in Lebanon, which remain to be a heavy burden within this population [23].

### Care-seeking and associated medication use among Syrian refugees with NCDs in Lebanon

Given the large caseload of Syrian refugees with NCDs in Lebanon coupled with the high costs of providing NCD care, implications on the Lebanese healthcare system are substantial [32]. The MoPH and the UNHCR undertook measures in response to the Syrian crisis to provide primary healthcare services for Syrian refugees through the primary healthcare centers (PHCs) across the country’s governorates at subsidized costs [23, 24, 33]. The UNHCR pays 75% or up to 100% of hospitalization costs for the most vulnerable refugees and for those who need life-saving [6]. However, due to limited funding, the UNHCR has insufficient capacities to provide health coverage for chronic conditions such as renal failures, diabetes and certain cancers, except for life-threating cases [6, 34]. In parallel to the Lebanese MoPH system, international non-governmental agencies have been providing free-of-charge primary care for Syrian refugees with NCDs, including diabetes and hypertension, in both North Lebanon and in the Bekaa valley since early 2012 [33, 35], and in South of Beirut since 2013 [23].

Care-seeking for NCDs among Syrian Refugees in Lebanon reached up to 82.9% and was distributed as 88.2% seeking care for diabetes, 82.6% for CVDs and 80.9% for hypertension over a period of 4 years from the beginning of the Syrian crisis in 2011 [24]. Comparable findings were reported with regards to NCD-associated medication use [36, 37]; however, interval discontinuation of medications was documented [11, 38]. Despite the reportedly high rates of healthcare seeking to manage their NCDs, Syrian refugees in Lebanon declared that they faced complications [23]. The primary obstacle was the costs of NCDs treatment. It was stated that 33–77% of Syrian refugees in Lebanon suspended NCDs treatment due to high costs [37] coupled with other barriers that involved transportation costs, limited capacities of healthcare facilities, and suboptimal NCDs health education. Only 39% of Syrian refugees in Lebanon reported attending healthcare facilities or mobile clinics of local NGOs to receive NCDs health education [23].

The number of studies evaluating the prevalence of NCDs among Syrian refugees in Lebanon is on the rise; however, literature still lacks a country-wide study with a representative large sample size of Syrian refugees through which prevalence of NCDs can be widely studied. Hence, this study aims to determine the prevalence of NCDs among adult Syrian refugees in Lebanon, with a focus on hypertension, diabetes, CVDs and cancer. The study also aims to explore factors potentially related to the prevalence figures and understand the medication use associated with these morbidities.

## Methods

### Study design and overview of the Sijilli database

This study is a secondary analysis of de-identified data from the ‘Sijilli Electronic Health Records for Refugees’ Database which comprises data on 10,082 Syrian refugees in Lebanon [39]. This database was created between July 2018 and January 2020; the health information included in the Sijilli database is originally provided by the refugees and primarily collected by teams of medical professionals led by staff at the Global Health Institute of the American University of Beirut. The team conducted one-time visits to refugees with the aim to establish their portable Sijilli HER while in their settlement. Data collection took place in different refugees’ informal tented settlements across Lebanon covering all four locations adopted by UNHCR for data reporting (Bekaa, North Lebanon, Beirut/Mount Lebanon, and South Lebanon). The Sijilli EHR Database was capped at 10,082 all-age patients. Therefore, the sample size in each of these locations was proportionate to the overall Syrian refugee population residing in the latter based on UNHCR data [40]. The data records were distributed as such: 3565 refugee records (35.4%) from Bekaa, 2657 refugee records (26.4%) from North Lebanon, 2146 refugee records (21.3%) from Beirut, and 1714 refugee records (17.0%) from South Lebanon. The choice of refugees who benefited from the Sijilli EHR was random whereby the team would announce the date of visits and the interested refugees would show up. There was no discrimination in terms of gender, age, or disability. The Sijilli EHR Database covered self- reported data divided into seven sections; these are: socio-demographic information, social and lifestyle habits, medical and surgical history, OBGYN conditions, current medication use (at the time of data collection), vaccination history, and mental health screening. The socio-demographic section includes basic socio-demographic information such as age, gender, Syrian city of origin, location of the settlement, and year of migration to Lebanon. Risk factors were identified through data from the social and lifestyle section that addressed smoking, alcohol drinking, and physical exercise. The collected data is self-reported and not clinically validated by a nurse or a doctor, or biologically validated by a blood pressure or random blood glucose measurement or any other type of blood test at the time of the data collection interview with the refugees. Data on morbidities was extracted from medical and surgical history section that followed the International Classification of Diseases (ICD) 10 by the World Health Organization to report conditions [41]. Medication use was deduced from the medications section in the Sijilli EHR Database, which reported namely chronic medications. Categorization of medications was based on Davis’s Drug Guide for Nurses, which classifies medications according to their type [42].

### Inclusion criteria

All records of refugees aged 18 years or above were eligible for inclusion. Only those who had hypertension, diabetes, CVDs, or cancer reported as a morbidity in the medical conditions section were included in the analysis. The year 2011, given that the Syrian conflict that triggered the massive migration started back then, was used as reference year for move date.

### Data analysis

Data collected were coded and exported to SPSS v26 [43]. The prevalence of each diagnosis of NCD including: hypertension, diabetes, CVDs and cancer, taking into consideration the multi-morbidity were calculated. We then calculated the distribution of potential risk factors by NCD diagnosis and any differences in their distribution between diagnoses is present using frequency and percentages and tested for statistical significance using the Pearson’s Chi-square. These risk factors included: age groups, gender, smoking and alcohol intake status, and physical activity. Dates of birth, and date of displacement to Lebanon were used to calculate age and period of displacement. Average years of displacements per disorders were tested using the independent t-test. We evaluated the current and most frequent medication used by the refugees. The medications were identified, tabulated, and tested for association with hypertension, diabetes, and CVDs using the Pearson’s Chi-square. Multivariate logistic regressions were built to assess independent effect of factors on the prevalence of the three major disorders. Coefficients and Standard errors were exponentiated to create odds ratios, and 95% confidence interval. All analyses were run at the 0.05 statistical significance level.

## Results

### NCDs prevalence by sample characteristics

Out of 3255 records of Syrian refugees aged 18 years or older, 523 refugees (16.0%) reported having at least one of the following conditions: hypertension, diabetes, CVDs or cancer (Table 1). The majority reported having hypertension (10.0%), while diabetes was the second most reported NCD (5.7%), followed by CVDs (5.4%) and cancer (0.6%). The prevalence of all investigated NCDs increased significantly with age (*p* < 0.001). Males reported significantly higher prevalence (7.0%) of CVDs compared to females (4.6%). Former smokers had significantly higher prevalence of hypertension (24.2%; *p* < 0.001), diabetes (11.0%; *p* = 0.048), and CVDs (18.7%; *p* < 0.001). On the other hand, former drinkers had significantly higher CVDs prevalence (22.2%; *p* = 0.048), compared to current drinkers who had higher cancer prevalence (5.9%; *p* = 0.013). For adult refugees who moved to Lebanon after 2011, hypertension was the only NCD who showed significant higher mean of years spent in Lebanon with the disease (M = 5.1, SD = 2.1) compared to those spent without it.

**Table 1:** Prevalence of Non-communicable Diseases (NCDs) among Population Sub-groups

Variable/disease	Hypertension N (%)	Diabetes N (%)	CVDs N (%)	Cancer N (%)
Prevalence (N = 3255)	326 (10.0%)	185 (5.7%)	175 (5.4%)	20 (0.6%)
95% CI	0.09–0.11	0.05–0.06	0.05–0.06	0.00–0.01
*Age (years)*				
18–39 (N = 2066)	57 (2.8%)	22 (1.1%)	46 (2.2%)	11 (0.5%)
40–59 (N = 959)	169 (17.6%)	111 (11.6%)	82 (8.6%)	7 (0.7%)
60 + (N = 230)	100 (43.5%)	52 (22.6%)	47 (20.4%)	2 (0.9%)
*p*-value	< .001	< .001	< .001	
*Sex*				
Male (N = 1071)	97 (9.1%)	63 (5.6%)	75 (7.0%)	3 (0.3%)
Female (N = 2184)	229 (10.5%)	122 (5.9%)	100 (4.6%)	17 (0.8%)
*p*-value	.202	.732	.004	.087
*Smoking status*				
Never smoker (N = 2109)	197 (9.3%)	111 (5.3%)	94 (4.5%)	13 (0.6%)
Current smoker (N = 920)	95 (10.3%)	58 (6.3%)	57 (6.2%)	5 (0.5%)
Former smoker (N = 91)	22 (24.2%)	10 (11.0%)	17 (18.7%)	1 (1.1%)
Missing	12	6	7	1
*p-*value	< .001	.048	< .001	.807
*Alcohol intake*				
Never drinker (N = 2983)	299 (10.0%)	177 (5.9%)	158 (5.3%)	16 (0.5%)
Current drinker (N = 17)	2 (11.8%)	0 (0.0%)	0 (0.0%)	1 (5.9%)
Former drinker (N = 9)	3 (33.3%)	1 (11.1%)	2 (22.2%)	0 (0.0%)
Missing	22	7	15	3
*p*-value	.067	.471	.048	.013
*Physical activity*				
No (N = 2328)	236 (10.1%)	131 (5.6%)	113 (4.9%)	15 (0.6%)
Yes (N = 611)	56 (9.2%)	35 (5.7%)	38 (6.2%)	1 (0.2%)
Missing	34	19	14	4
*p*-value	.475	.923	.174	.151
*Years spent in Lebanon (*> *2011)*				
Mean (SD)				
With disease	5.1 (2.1)	5.0 (2.1)	4.8 (2.1)	5.2 (1.9)
Without disease	4.8 (2.1)	4.8 (2.1)	4.8 (2.1)	4.8 (2.1)
*p*-value	.020	.205	.807	.393

### Prevalence of NCD comorbidities

**Fig. 1:**
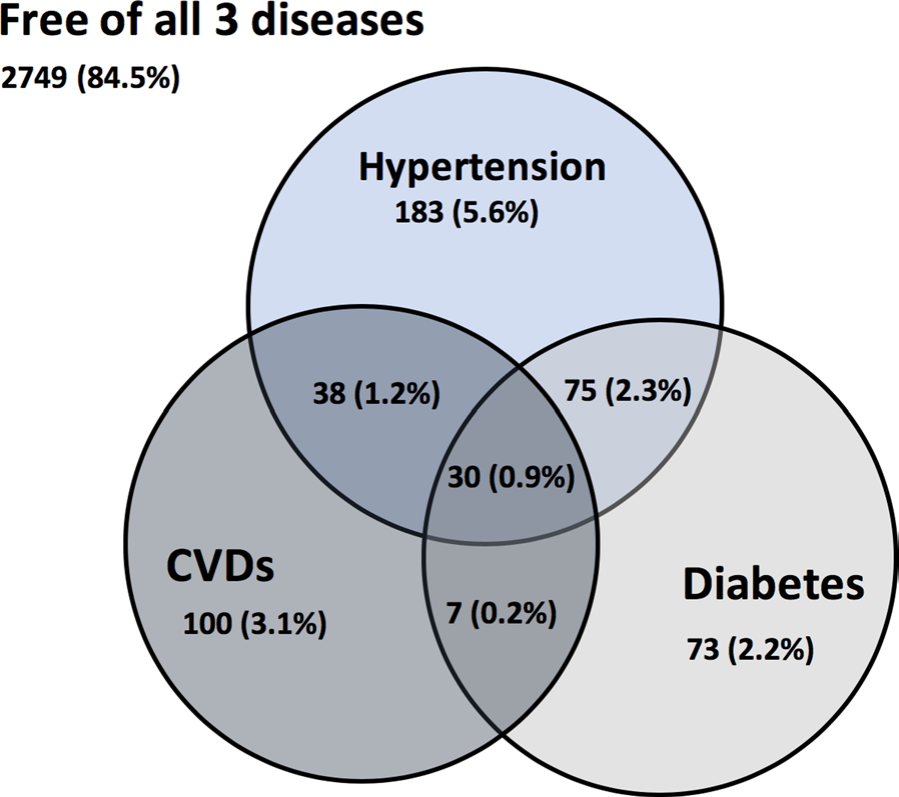
Visual representation of the prevalence of hypertension, diabetes, and CVDs among adult Syrian refugees, and the overlap among these conditions indicating the presence of comorbidities

Presence of comorbidities was explored among hypertensive, diabetic and adult refugees with CVDs, excluding adults with cancer. A total of 506 (15.5%) adult refugees reported at least one of the three conditions, while 2749 (84.5%) were free of the three conditions (Fig. 1). Of those who reported supporting from the NCDs of our interest, 371 (11.4%) refugees reported having 1 condition, 121 (3.70%) reported having 2 conditions, and 31 (1.0%) reported having 3 or more conditions.

**Fig. 2:**
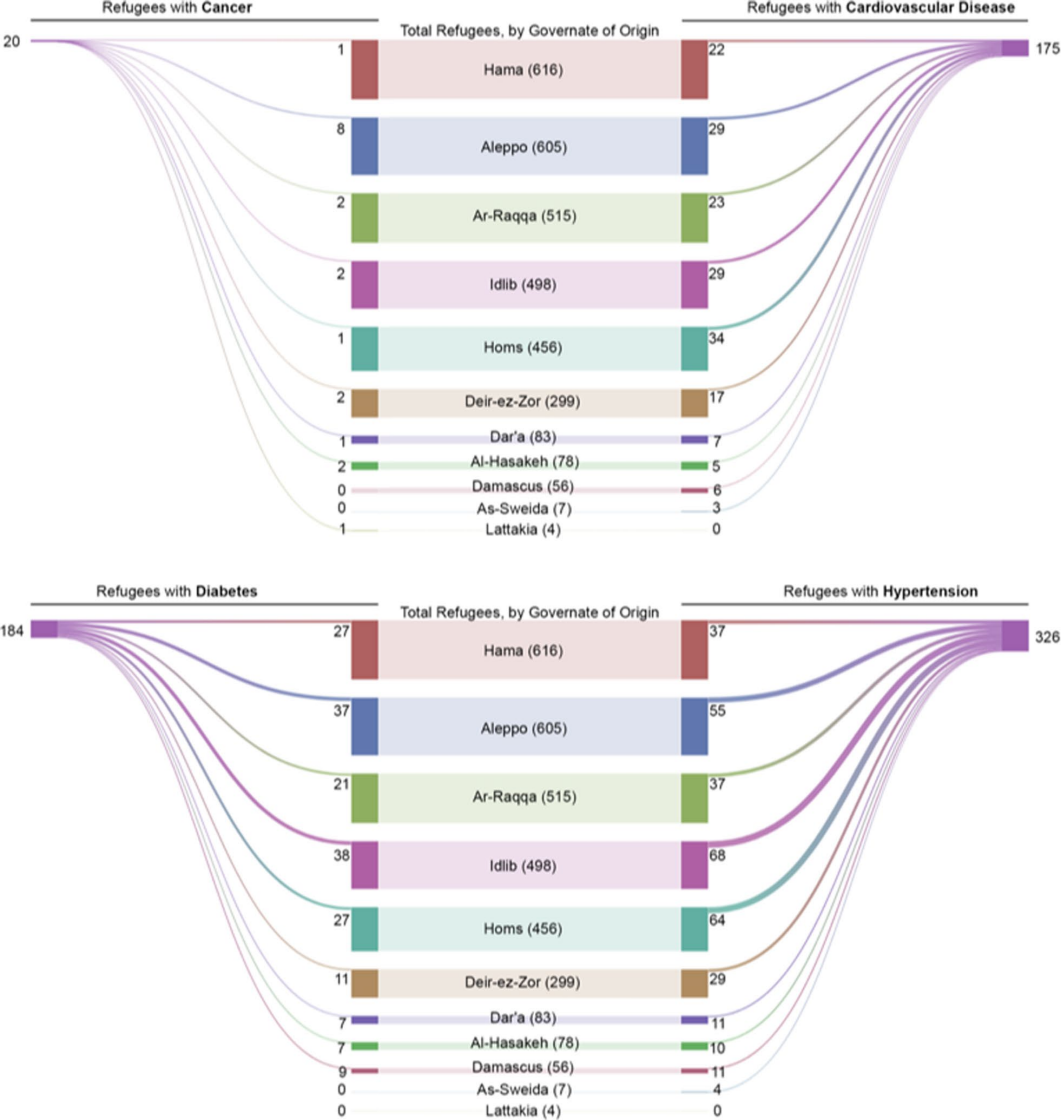
Visual representation of the governorate of origin of adult refugees with hypertension, diabetes, CVDs, or cancer

Figure 2 presents the governorate of origin of adult refugees reporting to have hypertension, diabetes, CVDs or cancer. Aleppo, Idlib, and Homs were noted to be the main three governorates of origin for refugees with hypertension, diabetes or hypertension, while multiple governorates were reported as the main origins of refugees with cancer.

### Medication use

Analysis was performed to understand the characteristics of medication-use among adult refugees. Table 2 shows that the majority of the adult refugees having either hypertension or diabetes take corresponding medications (59.5% and 67.0% respectively), with the majority taking one medication for their respective condition. For CVDs on the other hand, the proportion of those who take medication (49.1%) is very similar to those who don’t (50.9%), with the majority of the former taking more than one medication (28.0%). A significantly higher proportion of female adult refugees who were diagnosed with hypertension before moving to Lebanon, were on medication (78.9%) compared to those diagnosed after moving to Lebanon (59.7%). No differences were reported for female adult refugees with diabetes and CVDs with regards to medication use based on diagnosis date/location. On the other hand, a higher proportion of male adult refugees who were diagnosed with diabetes after moving to Lebanon were on medication compared to those diagnosed in Syria prior to displacement.

**Table 2:** Characteristics of medication use of adult Syrian refugees reporting hypertension, diabetes, and cardiovascular diseases (CVDs)

	Hypertension (N = 326)		Diabetes (N = 185)		CVDs (N = 175)	
	N	%	N	%	N	%
*Taking medication*						
No	132	40.5%	61	33.0%	89	50.9%
Yes	194	59.5%	124	67.0%	86	49.1%
*Number of medications*						
1	152	46.6%	95	51.4%	37	21.1%
2	29	8.9%	26	14.1%	18	10.3%
3 +	13	4.0%	3	1.6%	31	17.7%
*Females taking medication*						
Among those diagnosed before moving to Lebanon	45 (N = 57)	78.9%	28 (N = 32)	87.5%	9 (N = 18)	50.0%
Among those diagnosed after moving to Lebanon	46 (N = 77)	59.7%	29 (N = 46)	63.0%	14 (N = 33)	42.4%
Date of move is unknown	(N = 95)		(N = 44)		(N = 49)	
*p*-value	.019	.607	.603			
*Males taking medication*						
Among those diagnosed before moving to Lebanon	19 (N = 26)	73.1%	19 (N = 26)	73.1%	15 (N = 21)	71.4%
Among those diagnosed after moving to Lebanon	20 (N = 32)	62.5%	17 (N = 20)	85.0%	19 (N = 27)	70.4%
Date of move is unknown	(N = 39)		(N = 17)		(N = 27)	
*p*-value	.393	.038	.936			

Table 3 presents the current medication use among adult refugees reporting to have at least one condition of the following: hypertension, diabetes, or CVDs. Data on medication use indicates that beta-blockers are the most used drug class among hypertensive adult refugees generally (17.2%) and among those reporting only having hypertension as NCD. Similarly, biguanides were noted to be the most used medications among diabetic adult refugees generally (35.1%) even when diabetes is the only NCD reported. On the other hand, salicylates were noted to be the most used medications among adult refugees with CVDs alone and in combination with other NCDs such as hypertension.

**Table 3:** Most frequently used type(s) of medication by condition

Conditions	Top medication if taking 1 drug	N	%	Top medication in general	N	%
All hypertension	Beta-blocker	31	20.4	Beta-blocker	56	17.2
All diabetes	Biguanide	37	38.5	Biguanide	65	35.1
All CVDs	Salicylate	20	54.1	Salicylate	59	33.7
Only hypertension	Beta-blocker	12	15.0	Beta-blocker	23	12.6
Only diabetes	Biguanide	16	41.0	Biguanide	25	34.2
Only CVDs	Salicylate	7	66.7	Salicylate	26	26.0
All NCDs	N/A			Salicylate	13	43.3
Hypertension and diabetes	Biguanide	3	30.0	Biguanide	24	32.0
Hypertension & CVDs	Salicylate	1	20.0	Salicylate	16	42.1
Diabetes and CVDs	N/A			Biguanide and Salicylate	4	57.1

### Factors associated with NCDs among Syrian refugees

The Logistic Regression for Hypertension, Diabetes and CVDs based on baseline characteristics showed that being a female was associated with higher odds of having hypertension (OR = 1.55, *p* = 0.034), but rather lower odds of having CVDs with borderline significance (OR = 0.62, *p* = 0.05) (Table 4). Older age groups were at a significantly higher odds of being hypertensive, diabetic, or suffering from CVDs (*p* < 0.001). Higher odds of CVDs were found in former smokers with borderline significance noted (OR = 2.30, *p* = 0.052). A higher odds of being hypertensive was observed in those who have been longer in Lebanon (OR = 1.070; *p* = 0.037). Moving to Lebanon on the year 2011 or after was associated with higher odds of being hypertensive (OR = 3.715; *p* = 0.032) or diabetic (OR = 11.54; *p* = 0.035).

**Table 4:** Multivariate Logistic Regression Model for Hypertension, Diabetes and CVDs by Sample Characteristics. All variables are adjusted (i.e., all variables are in one model)

	Hypertension				Diabetes				CVDs			
	OR	95% CI		*p*-value	OR	95% CI		*p*-value	OR	95% CI		*p*- value
*Gender*												
Male	1.00	–	–		1.00	–	–		1.00	–	–	
Female	1.55	1.03	2.33	.034	1.00	0.62	1.60	0.998	0.62	0.38	1.00	0.050
*Age groups (years)*												
18–39	1.00	–	–		1.00	–	–		1.00	–	–	
40–59	6.79	4.41	10.47	< .001	8.32	4.60	15.02	< .001	4.52	2.60	7.88	< .001
60 +	30.33	18.10	50.83	< .001	22.59	11.61	43.95	< .001	11.61	6.11	22.07	< .001
*Smoking status*												
Never smoker	1.00	–	–		1.00	–	–		1.00	–	–	
Current smoker	0.99	0.67	1.47	0.968	0.89	0.55	1.43	0.625	0.84	0.50	1.41	0.510
Former smoker	2.02	0.95	4.30	0.070	1.05	0.41	2.73	0.916	2.30	0.99	5.32	0.052
*Alcohol intake*												
Never drinker	1.00	–	–		1.00	–	–		1.00	–	–	
Current drinker	0.90	0.04	18.59	0.946	*	*	*		*	*	*	
Former drinker	6.42	0.68	60.87	0.105	2.90	0.23	37.29	0.414	3.47	0.29	41.35	0.324
Length of stay in Lebanon (years)	1.070	1.00	1.14	.037	1.09	0.99	1.18	0.068	1.05	0.96	1.15	0.258
*Year moved to Lebanon*												
< 2011	1.00	–	–		1.00	–	–		1.00	–	–	
≥ 2011	3.715	1.11	12.34	.032	11.54	1.18	112.59	.035	3.200	.611	16.755	.168

^*^Cell numbers too few to produce estimate

## Discussion

### Prevalence of NCDs

This study examined the prevalence of hypertension, diabetes, CVDs and cancer among Syrian refugees settled in refugees’ informal tented settlements across Lebanon. It also shed light on medication-use patterns associated with these NCDs.

Our study revealed that hypertension was the most prevalent (10.0%) among the included NCDs among Syrian refugees in Lebanon. Prevalence of hypertension in our study are comparable to those (7.4%) reported by a study conducted among Syrian refugee adults in Lebanon by Doocy et al. in 2016 [24], but lower than estimates reported by other sources [11, 15, 23, 44]. Diabetes was the second highest prevalent condition (5.7%) after hypertension in our study. These findings are congruent with a study conducted among Syrian refugee adults in Lebanon [11]; however, discordant prevalence were observed in other studies ranging between 3.3 and 47% [11, 23, 24, 45]. With regards to other conditions, the prevalence of CVDs in our study was 5.4%, consistent with results (3.3%) reported by others [24]. On the other hand, cancer prevalence was very low (0.6%); slightly lower than UNHCR survey estimates (2%) among Syrian refugees in Lebanon [15].

Discrepancies in the prevalence reported in our study and those reported elsewhere in the literature could be explained in different ways. One possible explanation could be linked to the location of refugees whether in camp or non-camp settings and the associated differences in the levels of access to healthcare. In addition, the implications of different definitions between different studies may be contribute to discrepancies in prevalence among the studies. For example, the prevalence hypertension, diabetes and other NCDs were calculated based on self-reported data through surveys and interviews of refugees reporting their diseases by-themselves hence, recall bias may be obtained as well as the under-presentation of undiagnosed cases. On the other hand, other studies reported the prevalence of NCDs based on medical records and e-health systems. The variations in the availability of interventional programs across refugees’ informal tented settlements targeted to control NCDs could provide additional explanation. Moreover, the dissimilarities observed in prevalence could be related to underreported cases, which reflect poor access to healthcare leading to undiagnosed hidden cases. Our research results indicate that efforts should be made to adopt low-cost, innovative methods to detect and control NCDs among refugees. This can be made for example through collaboration of the ministry of public health, the UNHCR, NGOs as well as public and private hospitals to ensure an equal access to healthcare services approached through mobile clinics, e-health services, weekly camp visits of medical practitioners under training with experienced supervision.

Looking at the non-modifiable risk factors for NCDs, we found that higher age was associated with higher NCDs prevalence, consistent with previous findings [24, 46]. This is further supported by evidence suggesting that susceptibility to NCDs increases with age [47]. Regarding gender-related prevalence of NCDs, our study findings indicate that both genders reported comparable prevalence of hypertension, diabetes and cancer. Males, however, reported significantly higher prevalence of CVDs compared to females. Although the incidence of CVDs in males is higher than in females [48], the prevalence of CVDs in females could be underestimated based on the misperception that females are usually protected from CVDs [49]. In addition, the neglect of attention to CVDs among Syrian refugee females could explain the observed variations in prevalence.

In terms of modifiable risk factors for NCDs, a strong linkage has been reported between smoking status and alcohol intake, and increased risk for NCDs [50]. This concurs with our findings, where former smokers were recorded to have significantly higher prevalence of hypertension (24.2%), diabetes (11.0%), and CVDs (18.7%) compared to current smokers and never-smokers. A potential explanation of our findings could be that these individuals may have reached high-risk or advanced stages of these NCDs that necessitated them to quit smoking, or alternatively became aware that smoking could aggravate their health status and decided to quit. While there was no association between physical activity and prevalence of NCDs for all conditions in our study, a strong correlation between low physical activity and increased risk for NCDs is reported in literature [51]. The lack of association between physical activity and NCDs prevalence in the present study may be due to the low prevalence of physical activity amongst the study population. Nonetheless, obtained results in this study and the reported associations of some risk factors and NCDs prevalence should be handled with caution because of the nature of data collection which is self-reported data by the refugees themselves rather than extraction of data from an e-healtn system of presence of any biological approve to confirm the diagnosis.

It was remarkable in our study that refugees originating from specific regions had tendency to have specific comorbidities. This observation was previously highlighted in literature stating that the prevalence of NCDs such as hypertension and diabetes is affected by refugees’ region of origin [52]. Our study revealed that the refugees with hypertension, diabetes and/or CVDs were originally from Idlib, Aleppo or Homs. This could relate to the fact that health systems in these governorates were significantly damaged as a result of the Syrian conflict [53]. Around 80% of health facilities in Idlib, and 60% of health facilities in Aleppo were completely or partially damaged as of February 2017. This, in turn, influenced the capacities of these health systems to deal with the increasing burden of NCDs [53]. The Syrian conflict has also led to a shortage of healthcare professionals who were forced to leave, and to a shortage in supply of medical equipment and medications [53]. All these factors could have contributed to the high prevalence of NCDs in these governorates, translated into a high burden of NCDs among refugees originating from these governorates. Furthermore, cancer cases in our study were mostly reported among Aleppian refugees, which is in line with previous reports indicating that cancer cases are mostly located in Aleppo among other governorates of Syria [54].

### Medication use patterns

Regular medication use may be challenging in the context of displacement [55]. Literature has shown disparities in the findings. Nearly 75% of Syrian refugees with an NCD in Lebanon reported taking their prescribed medications [36, 37]. Other indications suggested that 56.1% of Syrian refugee households in Lebanon had a member with an NCD that was unable to access medications [45]. Our findings indicate that access to medications at the time of the data collection was relatively high for hypertension (59.5%) and diabetes (67.0%). This is in agreement with a previous study conducted among Syrian refugees in Lebanon [32]. However, the latter reported interval interruptions in medication use for both conditions due to financial constraints [11, 38]. In our study, reasons for the low prevalence of medication-use for all conditions could be in line with previous reports from the literature. Inability to afford the high costs of medications in Lebanon compared to the pre-conflict costs in Syria was the main reported causative factor for the low ability to access needed medications [11, 38]. Lack of knowledge of where to buy medications and physical disabilities which limit movement provide other potential contributing factors [11]. This implies that hypertension and diabetes has a better chance of being controlled due to the high prevalence to medication-use by the refugees once the drugs are within their reach. Yet, further studies about the compliance rates should be initiated among the Syrian refugees in Lebanon and the region. This may imply longitudinal studies rather than database based on one interview with the participants. Hence, medical campaigns and awareness of NCDs and their complications should be a major focus in the future for healthcare systems that are concerned in treating refugees. Although Syrian refugees suffer from an immense burden of cancer, the latter are more difficult to diagnose through primary care centers alone as compared to other NCDs. Unfortunately, Syrians are ineligible for public health coverage in Lebanon and have to purchase private insurance to receive coverage. With limited funding, the UNHCR has been unable to provide coverage for complex cases such as cancer, except for particular life-threatening situations that are considered on a case-by-case basis [34]. This explains the low cancer prevalence obtained in our study and other studies in turn due to its underestimation. Thus, an increase in cancer awareness as well as investing in primary care services for cancer screening could prompt earlier diagnosis and treatment.

Study analysis revealed an association between medication use and location of diagnosis, which relatively differed by gender. For all three NCDs, females who were diagnosed before moving to Lebanon were more likely to take corresponding medications compared to those diagnosed in Lebanon, however, this was not the case among males. A higher proportion of male adult refugees who were diagnosed with diabetes after moving to Lebanon were actually on medication compared to those diagnosed in Syria prior to displacement. This observed gender gap in medication use based on the migration journey may be rooted in existing gender dynamics within refugee communities where men’s/husbands’ health may be often prioritized over women’s/wives’ health given that the husband is perceived as the primary bread winner of the family. Therefore, investing in buying a medication is more worth when it is for the male, while other conflicting priorities may emerge vis-à-vis buying a medication for the female.The findings from this study also demonstrated that the patterns of medication use for all conditions with or without comorbidities abided by the general recommended guidelines [56–58]. Consistent with our findings, beta-blockers and biguanides are the most frequently prescribed medications for hypertensive patients [56] and for diabetics [57], respectively, while salicylates are among the top medications used for patients with CVDs [58].

### Strengths and limitations

The present study has several strengths. One of its strengths lies in its large sample size. In addition, the study draws on data from refugees’ informal tented settlements in different areas in Lebanon; the sample population is therefore representative of Syrian refugees in Lebanon. Furthermore, assessing disease prevalence involved the inclusion of factors known to influence the outcome measure such as region of origin, social and lifestyle behaviors, comorbidities, and number and medication use patterns. The results of this study should however be considered in light of certain limitations. First, the Sijilli EHR Database mainly relies on self-reported data for the variables of interest to the EHR which could have introduced some bias especially that there was no clinical verification and no biological verification. In addition, an underestimation of the NCDs conditions may have taken place, given that the Sijilli Database might have missed reporting on cases who don't know their diagnosis or have never been diagnosed. Second, self-reported medication use may be associated with recall bias. Additionally, the study does not address the concept of care-seeking behavior as this was beyond its scope. The cross-sectional design also adds an additional layer of complexity when it comes to interpreting the associations identified such as the gender gap in taking medications taking into consideration the migration journey. Last, although the sampling in the Sijilli database is reported as random with all refugees residing in the visited settlement being eligible for inclusion, yet there may be a potential selection bias given that some refugees would not have been able to show up at the day of data collection for some reasons (e.g. being at work, visiting a relative, etc.). Nevertheless, the data collection teams performed visits that spanned across mornings and afternoons to be able to capture those who may have been at work among others. Also, the sample size in each of the locations where data collection took place was proportionate to the overall Syrian refugee population residing in the settlments based on UNHCR data, which makes our sample relatively reflective of the characteristics of the overall Syrian refugees population in Lebanon.

## Conclusions

Nine years into the Syrian crisis, Lebanon has made remarkable efforts to respond to the healthcare needs of the Syrian population on its territories. However, providing high-quality NCDs services remains challenging in light of the high burden of NCDs that requires continuity of care, and the costly medications that are accompanied by funding shortfalls. Findings from this study suggest the possibility of many undiagnosed cases. Our findings suggest that efforts should be directed towards the employment of innovative low-cost approaches for NCD detection and control among refugees, with a focus on the importance of medication use. Such efforts remain imperative to control the increasing burden of NCDs amongst refugee populations and improve equitable access to NCD services.

## Data Availability

The datasets generated and/or analyzed during the current study are not publicly available due to the institutional ownership of data, but are available from the corresponding author on reasonable request.
